# Acute Respiratory Distress Syndrome after Onyx Embolization of Arteriovenous Malformation

**DOI:** 10.1155/2011/918185

**Published:** 2011-06-01

**Authors:** Isaac Tawil, Andrew P. Carlson, Christopher L. Taylor

**Affiliations:** ^1^Department of Surgery and Department of Emergency Medicine, University of New Mexico Health Sciences Center, The University of New Mexico, MSC 10 5610, Albuquerque, NM 87131-0001, USA; ^2^Department of Neurosurgery, University of New Mexico Health Sciences Center, The University of New Mexico, MSC 10 5615, Albuquerque, NM 87131-0001, USA

## Abstract

*Purpose*. We report a case of a 60-year-old male who underwent sequential Onyx embolizations of a cerebral arteriovenous malformation (AVM) which we implicate as the most likely etiology of subsequent acute respiratory distress syndrome (ARDS). *Methods*. Case report and literature review. *Results*. Shortly after the second Onyx embolization procedure, the patient declined from respiratory failure secondary to pulmonary edema. Clinical entities typically responsible for pulmonary edema including cardiac failure, renal failure, iatrogenic volume overload, negative-pressure pulmonary edema, and infectious etiologies were evaluated and excluded. The patient required mechanical ventilatory support for several days, delaying operative resection. The patient met clinical and radiographic criteria for ARDS. After excluding other etiologies of ARDS, we postulate that ARDS developed as a result of Onyx administration. The Onyx copolymer is dissolved in dimethyl sulfoxide (DMSO), a solvent excreted through the lungs and has been implicated in transient pulmonary side effects. Additionally, a direct toxic effect of the Onyx copolymer is postulated. *Conclusion*. Onyx embolization and DMSO toxicity are implicated as the etiology of ARDS given the lack of other inciting factors and the close temporal relationship. A strong physiologic rationale provides further support. Clinicians should consider this uncommon but important complication.

## 1. History and Presentation

**Figure 1:**
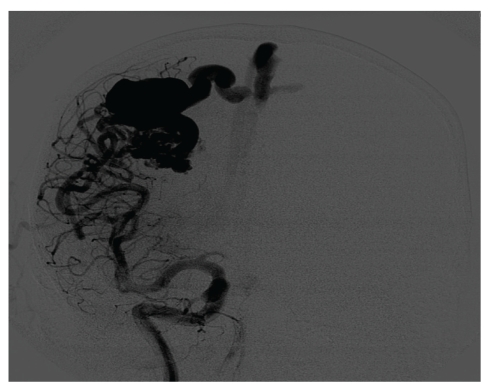
AP and lateral projections of angiogram of right selective internal carotid artery injection showing the arteriovenous malformation in this late arterial phase, with filling of a large venous channel into the superior sagittal sinus.

This 60-year-old male initially presented with a history of seizures and progressive left-sided hemiparesis. A 3 cm AVM was discovered in the right posterior frontal lobe, just anterior to the central sulcus (Figure 1). The patient's pastmedical history included hypertension, diet-controlled diabetes mellitus, and mild chronic obstructive pulmonary disease. Medications included levetiracetam for seizure control and metoprolol for hypertension. Given the progressive symptomatology, it was decided to perform an operative resection of the AVM after sequential Onyx embolization procedures.

## 2. Interventions and Hospital Course

On admission day one, the patient underwent the first of two preoperative embolization procedures. Both procedures were performed under general anesthesia with endotracheal intubation. Arteriography demonstrated feeding of the AVM via both middle cerebral artery (MCA) and anterior cerebral artery (ACA) branches, with early venous drainage via a large cortical vein near the entry into the superior sagittal sinus. An anterior feeding branch off the MCA was selectively catheterized using an UltraFlow microcatheter and Mirage microwire (Ev3 Neurovascular, Irvine, Calif, USA). When a position adjacent to the nidus was achieved, the Onyx was injected per the standard procedure. The dead space volume of the microcatheter was displaced with 0.26 mL DMSO. Onyx- 18 embolization of the prefrontal branch was performed using 0.8 mL at an injection rate of 0.1 mL/min until there was reflux into the feeding vessel. A second feeding vessel was then selectively catheterized, and due to the apparent higher flow rate, Onyx-34 was used. 1.6 mL of the Onyx was injected after the same DMSO flush. The Onyx appeared to fill the nidus well with no noted venous filling. Follow-up angiography demonstrated improvement in the transit time into the draining vein but some residual AVM. The patient was hemodynamically stable through the procedure and had no respiratory difficulties. He was extubated uneventfully and transferred to the neurosciences intensive care unit for monitoring and strict blood pressure control. 

**Figure 2:**
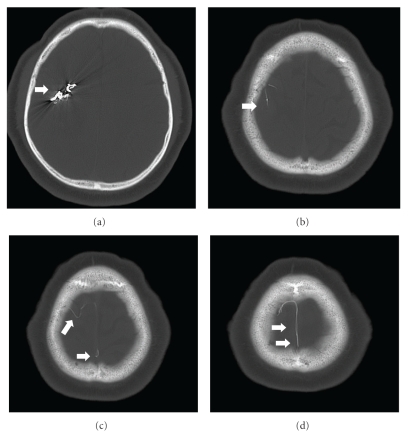
Bone windows of CT of the head after first embolization, showing high attenuation embolic material (Onyx) in the nidus of the AVM (a) as well as extension through the draining vein and a thin strand in the superior sagittal sinus.

 The immediate postembolization course was complicated by a brief self-limited seizure requiring only supportive care. The postprocedure CT scan revealed a small amount of subarachnoid hemorrhage in the right sylvian fissure adjacent to the AVM, embolization material within the AVM, and a small tail of Onyx extending through the draining vein into the superior sagittal sinus (Figure 2). The patient's neurological exam was otherwise unchanged. On postembolization day one, the patient had a mildly increased creatinine to 1.25 mg/dL, despite a positive fluid balance of 1.2 liters, which was attributed to the induced relative hypotension below the patient's baseline compounding a contrast nephropathy. He required increasing levels of supplemental oxygen via nasal cannula despite postprocedure spirometry and pulmonary toilet. 

**Figure 3:**
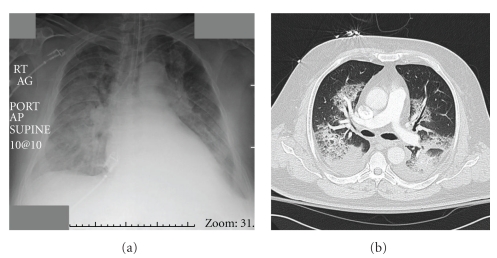
AP chest X-ray and accompanying select axial CT slice from the day of respiratory deterioration. The typical “ground glass” appearance and consolidation is noted.

 The following day, the patient was assumed to be clinically stable and so underwent a second embolization procedure. The anterior cerebral artery (ACA) was selectively catheterized, and using the same microcatheter as above, two separate feeding vessels to the AVM were identified and injected with Onyx for a total volume of 1.6 mL of Onyx-34 and 0.52 mL of DMSO to occupy the catheter dead space. Subsequent angiography demonstrated no further supply to the AVM nidus from the ACA but persistent filling via small, distal MCA branches. Venous drainage to the sagittal sinus and vein of Labbe remained patent. This second embolization procedure was well tolerated by the patient without any oxygenation difficulties throughout the case and an uneventful postprocedure extubation. Over the next 24 hours, however, the patient's respiratory function declined with an increased work of breathing ultimately mandating intubation and mechanical ventilatory support. A CT angiogram of the chest was then performed to evaluate for pulmonary emboli and elucidate other etiologies of respiratory failure. The CT imaging demonstrated perihilar and bibasilar ground glass opacities and bibasilar interlobar septal thickening with Kerley B-lines, all consistent with pulmonary edema (Figure 3). There were no vascular filling defects, excluding the diagnosis of visible pulmonary emboli. This pulmonary edema was confirmed as noncardiogenic in origin as echocardiography performed the same day demonstrated hyperdynamic ventricular function (ejection fraction of 70%) and wall motion, without valvular abnormalities or diastolic dysfunction. The patient was clinically euvolemic with a normal urine output and a stable serum creatinine after the second embolization procedure. The patient's gas exchange was poor with a PaO2 : FiO2 ratio of 64–71 on hospital days 3 and 4. Imaging and diagnostic criteria were consistent with a diagnosis of ARDS. The patient did not demonstrate any of the typical risk factors associated with ARDS, including pneumonia, aspiration, reperfusion pulmonary edema, sepsis, pancreatitis, drug overdose, or transfusion of blood products, leaving the most likely etiology being administration of Onyx during embolization procedures. The patient remained afebrile, and there was no evidence of infection throughout his course. 

 Supportive pulmonary and systemic care followed, including lung protective ventilatory strategies and minimization of infused crystalloid. Six days later, the patient's respiratory function had improved and the patient underwent a successful frontoparietal craniotomy and AVM resection. The patient was extubated shortly after and was discharged home in good condition after a sixteen-day hospitalization. 

## 3. Discussion

Since endovascular embolization of cerebral AVMs was first described in 1960, numerous agents have been used to occlude the nidus or feeding arteries, all with various pros and cons [1]. In 1990, Taki and colleagues reported the development and successful use of ethylene vinyl alcohol copolymer (EVAC) for AVM embolization [2]. The formulation of this copolymer, mixed in a dimethyl sulfoxide (DMSO) solvent and combined with micronized tantalum (a radio-opacifying agent), is now marketed as Onyx (EV3 Neurovascular, Irvine, Calif, USA) and is FDA approved for endovascular embolization of cerebral AVMs. Several case series of its use and clinical efficacy in this setting have since been reported [3–5]. Other observational series reporting efficacy using Onyx for endovascular treatment of aneurysms and dural arteriovenous fistulae are increasingly common [6–8]. While all of these reports evaluated cerebral outcomes and related complications, none addressed potential systemic complications related to Onyx. 

Systemic pulmonary complications related to cerebral Onyx embolization have been reported elsewhere and attributed to the toxicity of the copolymer's solvent, DMSO [9]. DMSO [(CH_3_)_2_SO] is soluble in aqueous and organic media making it an efficient solvent for water insoluble compounds. Thus, it has been used as a biochemical solvent and a vehicle for drug therapy. It is this property that facilitates dissipation of the DMSO in the blood stream while the Onyx copolymer forms a spongy occlusive cast in the injected artery. Its other properties as a cell-differentiating agent and a hydroxyl radical scavenger led to its various therapeutic applications for rheumatologic disorders, anticancer therapies, and dermatologic disorders [9]. It is perhaps these properties that also lead to less desirable side effects and its potential toxicity to blood vessels both at the site of injection and at other end organs. DMSO has been shown to evoke an inflammatory response, vasospasm, and endothelial necrosis following intra-arterial injection. It is important to note however that several investigations demonstrated these effects at higher volumes and infusion rates than typically used during cerebral Onyx embolization [10, 11]. Another etiology of potential pulmonary toxicity lies in the excretion of DMSO. While DMSO metabolites are primarily eliminated via the kidneys, some early excretion occurs through the lungs. 

 Mild pulmonary toxicity has been well documented and often manifests as transient and self-limited hypoxemia during the embolization procedure. A retrospective series of 38 Onyx embolization procedures documented transient oxygen desaturation during all cases, requiring supplemental oxygen, but otherwise of no clinical consequence [12]. Another series reported similar transient desaturation during DMSO infusion and Onyx administration in 17 of 46 (37%) AVM embolizations [13]. One recent case report documented noncardiogenic pulmonary edema after Onyx embolization of an AVM [14]. The DMSO solvent was implicated as the possible cause after other etiologies were ruled out. The patient's course of mechanical ventilatory support lasted 2 days while the pulmonary edema resolved. 

 Our patient experienced worsening pulmonary edema over the two-day-periembolization period. It is unclear if one of the two embolization procedures was the offender or if there was cumulative effect of the multiple Onyx injections. The fact that the patient had a small strand of Onyx visible in the superior sagittal sinus after the first embolization procedure suggests the possibility that the pulmonary vasculature was exposed to the DMSO solvent and even potentially Onyx microemboli, though no radiodense material was seen grossly on the chest CT. The patient had a persistent supplemental oxygen requirement after the first procedure which increased after the second one. The echocardiogram and ECG confirmed the absence of a cardiac etiology of the pulmonary edema. Renal failure was also ruled out as the patient's mild increase in creatinine did not result in oliguria or impaired solute clearance. Negative-pressure pulmonary edema was ruled out as our patient had no extubation difficulties nor was the protracted course of the pulmonary edema consistent with a negative pressure phenomenon. No aspirations of gastric contents were observed during the uneventful intubations for the procedures, nor infectious etiologies were confirmed. The final diagnosis for the pulmonary process of ARDS was therefore made. ARDS is a clinical diagnosis for which there exist three sets of diagnostic criteria used by various practitioners. The Lung Injury Score definition, American-European Consensus Conference definition, and the Delphi definition all have varied sensitivities and specificities for the diagnosis [15]. Our patient met all three sets of diagnostic criteria. The patient remained intubated for 6 more days as his pulmonary failure improved to the point where he tolerated operative AVM resection and was subsequently extubated. 

## 4. Conclusion

ARDS in this patient was likely related to Onyx embolization given the lack of other inciting factors and the close temporal relationship. Potential mechanisms include inflammatory and toxic effects of DMSO or a direct effect of the ethylene vinyl alcohol via microembolic showering. The precise pathophysiologic mechanism as well as the true incidence of such a complication are unknown. We encourage clinicians to consider this uncommon but significant complication. Endovascular neurosurgeons should adhere to the recommended volumes and injection rates of Onyx in hopes of minimizing this potential complication. Further studies of Onyx embolization should evaluate noncerebral outcomes in addition to neurologic efficacy.
